# Comparative Effects of Different Carbohydrate–Protein Formulations on Resistance Training Adaptations in Healthy Young Men

**DOI:** 10.3390/nu18213513

**Published:** 2026-10-25

**Authors:** Wanyuan Liang, Xiang Wang, Congyu Sun, Yan Chen, Ye Sun, Dong Wang, Jingyi Zhang, Chengnan Zhang, Bing Wei, Junqiang Qiu

**Affiliations:** 1 China Basketball College, Beijing Sport University, Beijing 100084, China; liangwanyuan@bsu.edu.cn (W.L.); wx021330@163.com (X.W.); 2 Department of Exercise Biochemistry, Exercise Science School, Beijing Sport University, Beijing 100084, China; ryannchen@bsu.edu.cn (Y.C.); sunye@bsu.edu.cn (Y.S.); zhangjngyi0796@bsu.edu.cn (J.Z.);; 3 Beijing Competitor Sports Nutrition Research Institute, Beijing 100029, China; 4 School of Education, Beijing Sport University, Beijing 100084, China; 5 Beijing Sports Nutrition Engineering Research Center, Beijing 100084, China

**Keywords:** resistance training, carbohydrate–protein supplementation, carbohydrate formulation, skeletal muscle adaptation, body composition

## Abstract

**Background/Objectives:** Evidence is limited on whether carbohydrate formulations enhance resistance training adaptations when protein intake is relatively adequate. This study compared two carbohydrate–protein formulations in healthy young men. **Methods:** In this randomized, double-blind, placebo-controlled trial, 36 untrained men aged 18–25 years were assigned to a maltodextrin-based carbohydrate control group (PLA), low-molecular-weight carbohydrate plus protein (LMW-CHO+PRO), or high-molecular-weight carbohydrate plus protein (HMW-CHO+PRO). Thirty-three participants completed 8 weeks of supervised resistance training three times weekly and daily supplementation (50 g). Body composition, muscle circumference and thickness, strength, and serum testosterone, cortisol, and testosterone-to-cortisol ratio were assessed before and after intervention. The trial was registered with the Chinese Clinical Trial Registry (ChiCTR2500110509). **Results:** Body mass, total muscle mass, muscle circumference and thickness, and strength improved in all groups. Both supplementation groups showed greater improvements than PLA across several muscular adaptation measures. Time × group interactions were significant for body mass and total muscle mass, but not fat mass. HMW-CHO+PRO produced greater increases in body mass and total muscle mass than LMW-CHO+PRO; however, no consistent differences between formulations were observed in regional muscle circumference, muscle thickness, strength, or blood biomarkers. **Conclusions:** Under conditions of relatively adequate protein intake, combined carbohydrate and protein supplementation may provide additional support for resistance training adaptations. These findings do not establish an overall advantage of either formulation, nor can the observed differences be attributed specifically to carbohydrate molecular weight.

## 1. Introduction

Resistance training is an effective strategy for promoting skeletal muscle hypertrophy, increasing muscular strength, and improving body composition [1,2,3]. Skeletal muscle mass and function are important not only for physical performance but also for metabolic health, physical function, and long-term health outcomes [4,5,6]. Regular resistance training induces muscular adaptations through stimuli such as mechanical tension, metabolic stress, and muscle fiber microdamage, which collectively activate pathways involved in muscle protein synthesis, promote increases in muscle fiber cross-sectional area, and improve neuromuscular function [7,8]. However, the magnitude of adaptation to resistance training is influenced by multiple factors, including the training stimulus, nutritional intake, and recovery status [9]. Among nutritional strategies, protein supplementation is widely recognized as an important means of supporting skeletal muscle adaptation by providing essential amino acids for muscle protein synthesis and facilitating gains in fat-free mass and muscular strength [10]. Once protein intake is sufficient to support training adaptations, however, further increases in protein intake may provide only limited additional benefit [11]. This raises an important question as to whether other nutritional factors can further support recovery and long-term adaptation to resistance training when protein intake is already adequate.

Carbohydrate is an important energy substrate during resistance exercise, contributing to the maintenance of training capacity, preservation of muscle glycogen stores, post-exercise glycogen resynthesis, and performance in subsequent training sessions [12,13,14]. Adequate carbohydrate availability may be particularly relevant when training volume or frequency is high, as it may help sustain training quality and recovery between sessions. Carbohydrate sources and formulations differ in molecular structure, digestion and absorption characteristics, glycemic responses, and gastrointestinal tolerance, and these differences may influence substrate availability during exercise and recovery after training [15,16]. Co-ingestion of carbohydrate and protein has also been reported to improve energy availability, stimulate insulin secretion, and facilitate post-exercise recovery [17,18]. However, when habitual protein intake is already sufficient, evidence remains inconclusive as to whether additional carbohydrate intake further enhances resistance training-induced gains in muscle mass and strength [19,20,21]. Previous studies have largely focused on protein supplementation alone, supplementation with a single carbohydrate source, or acute metabolic and recovery responses to exercise. Direct comparisons of different carbohydrate formulations during prolonged resistance training, particularly under conditions of relatively adequate protein intake, remain limited. Moreover, commercially available or mixed-carbohydrate formulations often differ simultaneously in carbohydrate source, composition, and physicochemical properties. Consequently, any differences in long-term training adaptations cannot be readily attributed to a single characteristic, such as carbohydrate molecular weight.

Against this background, we conducted an 8-week resistance-training study in healthy young men to compare changes in muscle mass, muscular strength, and body composition across two carbohydrate–protein formulations and a placebo condition. Participants received either an LMW-CHO+PRO formulation, in which maltodextrin, maltooligosaccharides, and glucose were the main carbohydrate sources; an HMW-CHO+PRO formulation containing a mixture of isomaltulose, highly branched cyclic dextrin, Vitargo^®^ modified starch, fructose, and other carbohydrate sources; or a placebo supplement (PLA) consisting primarily of maltodextrin. The maltodextrin-based control was used to approximate the energy content, dosage form, and sensory characteristics of the study supplements as closely as possible, thereby helping to maintain blinding and control for potential effects associated with the supplementation procedure itself. In addition, maltodextrin provided the control group with a standardized, rapidly available carbohydrate source, allowing the effects of the two mixed formulations to be evaluated against a common carbohydrate reference condition. The two active formulations provided comparable amounts of protein and differed primarily in their carbohydrate sources and composition.

The study was designed to examine whether, under conditions of relatively adequate overall protein intake, differences in carbohydrate formulation could further influence adaptations to resistance training. Importantly, the study was not intended to isolate the independent effect of carbohydrate molecular weight. Rather, we evaluated the overall effects of the two carbohydrate–protein formulations during prolonged resistance training, with the aim of providing experimental evidence to inform nutritional supplementation strategies for resistance-trained individuals.

## 2. Materials and Methods

### 2.1. Participants

Sample size was estimated using G*Power software (version 3.1.9.2; Heinrich-Heine-Universität Düsseldorf, Düsseldorf, Germany). The effect-size parameter was based on a previous study examining resistance training and lean body mass outcomes [22]. Assuming a Cohen’s *f* of 0.55, a two-sided significance level of *α* = 0.05, and a statistical power of 1 − *β* = 0.80, the calculation was performed using a fixed-effects one-way analysis of variance (ANOVA), consistent with the three-group parallel design and based on between-group differences in change scores for the primary outcome. The analysis indicated that at least 12 participants per group were required, corresponding to a total target sample of 36 participants.

Thirty-six healthy, untrained men aged 18–25 years were recruited through public advertisements and volunteered to participate. All volunteers underwent preliminary screening. Participants were eligible if they met the following criteria: (1) aged 18–25 years; (2) body mass index (BMI) within the normal range of 18.5–23.9 kg/m^2^; (3) no clinically diagnosed disease; (4) no smoking or excessive alcohol consumption; (5) able and willing to comply with the study protocol; and (6) no participation in any clinical, food, or nutritional supplementation study during the preceding month.

The study protocol was conducted in accordance with the Declaration of Helsinki and was approved by the Sports Science Experimental Ethics Committee of Beijing Sport University on 23 June 2025 (approval no. 2025292H). All participants were fully informed of the study procedures and potential risks and benefits before providing written informed consent. The trial was registered with the Chinese Clinical Trial Registry on 14 October 2025 (ChiCTR2500110509). Of the 36 participants initially enrolled, 33 completed the full study protocol. Three participants withdrew because of non-compliance with the prescribed dietary requirements (*n* = 2) or failure to consume the study supplement as instructed (*n* = 1).

Participant characteristics are presented in Table 1. At baseline, no significant between-group differences were observed in age, height, body mass, or other basic physiological characteristics.

### 2.2. Study Design and Randomization

This 8-week study was conducted as a randomized, double-blind, controlled, parallel-group trial comprising a pre-screening phase and an intervention phase. During pre-screening, participants completed self-reported dietary and lifestyle questionnaires and provided 3-day dietary records, including two weekdays. Individuals who met the eligibility criteria were enrolled in the study.

Following baseline assessments, eligible participants were assigned a unique identification number and randomly allocated in a 1:1:1 ratio to the low-molecular-weight carbohydrate plus protein group (LMW-CHO+PRO), the high-molecular-weight carbohydrate plus protein group (HMW-CHO+PRO), or the maltodextrin-based carbohydrate control group (PLA). The randomization sequence was generated using Microsoft Excel. Participant identification numbers were entered in the first column, and random numbers were generated using the RAND() function. The generated random numbers were then copied as fixed values and sorted in ascending order. Participants in the first third of the randomized sequence were assigned to the LMW-CHO+PRO group, those in the middle third to the HMW-CHO+PRO group, and those in the final third to the PLA group. Randomization and treatment coding were performed by personnel who were not involved in intervention delivery, outcome assessment, or data analysis. Researchers responsible for intervention implementation and data collection had no access to the treatment allocation and identified participants only by their study codes. Outcome assessors and statistical analysts remained blinded to group identity until completion of the analyses. Following baseline assessments, participants were randomly allocated to one of three groups: the low-molecular-weight carbohydrate plus protein group (LMW-CHO+PRO), the high-molecular-weight carbohydrate plus protein group (HMW-CHO+PRO), or the isoenergetic placebo group (PLA). Group allocation was performed by personnel independent of the research team, and both participants and investigators remained blinded to treatment assignment.

**Figure 1:**
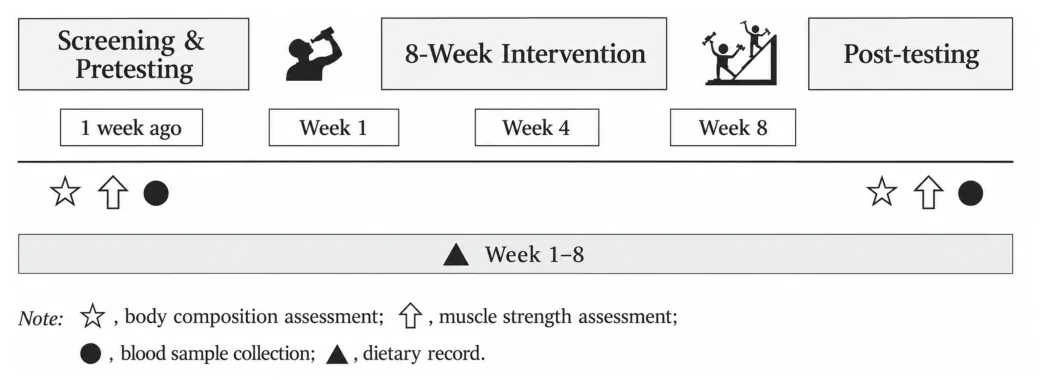
Study flow diagram.

All participants completed a supervised resistance-training program three times per week for 8 weeks. One-repetition maximum (1RM) testing was conducted at weeks 4 and 8. Throughout the intervention, participants were instructed to maintain their habitual diet and lifestyle and were provided with dietary guidance to promote consistency. Standardized dietary instructions were provided before each testing session, and participants consumed a standardized breakfast on testing days. Anthropometric and body-composition assessments were performed at baseline and after the 8-week intervention, with blood samples collected at both time points for biochemical analyses. The study timeline is shown in Figure 1.

### 2.3. Supplementation Protocol

Participants were assigned to one of three supplementation groups: a low-molecular-weight carbohydrate plus protein group (LMW-CHO+PRO), a high-molecular-weight carbohydrate plus protein group (HMW-CHO+PRO), or a placebo group (PLA). All supplements were provided in powdered form and were prepackaged and distributed according to randomization codes by personnel who were not involved in training supervision, outcome assessment, or statistical analysis. Supplements were supplied in identical opaque packages and uniformly flavored to minimize differences in appearance, odor, and taste. Both participants and investigators remained blinded to group allocation throughout the intervention and outcome assessment, and treatment codes were revealed only after completion of the statistical analyses.

During the 8-week intervention, participants in the LMW-CHO+PRO and HMW-CHO+PRO groups consumed one 50 g serving of the assigned nutritional supplement daily. Participants in the PLA group consumed one 50 g serving of placebo per day, consisting primarily of maltodextrin. Each serving was dissolved in 250 mL of room-temperature water and consumed in a single dose. On training days, the supplement was consumed within 30 min after completion of the resistance-training session; on non-training days, it was consumed with breakfast after waking. The nutritional composition and principal ingredients of each supplement are presented in Table 2 and Table 3, respectively.

Supplements were prepared in advance according to participant identification numbers and distributed by the research staff. Adherence was monitored throughout the intervention. After each scheduled intake, participants were required to photograph the consumed supplement and upload the image to a designated messaging group. The research team reviewed these records to verify and document supplementation compliance. Supplement adherence was calculated as the number of completed supplementation occasions divided by the number of scheduled occasions × 100%. Each participant was scheduled to complete 56 supplementation occasions over the 8-week intervention. Adherence was considered acceptable when at least 48 supplementation occasions were completed, corresponding to an adherence rate of ≥85%. Throughout the study, participants were instructed not to consume any additional protein supplements or other sports nutrition products that could potentially influence training adaptations, thereby minimizing nutritional confounding during the intervention.

### 2.4. Resistance Training Protocol

All participants completed resistance training at 75–85% of their one-repetition maximum (1RM), three times per week for 8 weeks, with each session lasting approximately 90 min. Details of the training program are provided in Table 4. Each session consisted of a 10 min warm-up, approximately 70 min of resistance exercise, and a 10 min cool-down, with 2–3 min of rest between sets. The program targeted the major muscle groups of the chest, shoulders, arms, back, abdomen, and lower body.

Training intensity was progressively increased over the intervention. Participants trained at 75% of 1RM during weeks 1–4 and at 85% of 1RM from weeks 5–8, based on the preceding strength assessment. All training sessions were supervised by qualified coaches to ensure proper exercise technique and consistency in program implementation. Attendance, training loads, and completion of each training session were recorded by the research staff throughout the intervention. Training adherence was calculated as the number of completed training sessions divided by the number of scheduled sessions × 100%. Participants who completed at least 21 training sessions, corresponding to an adherence rate of ≥85%, and completed the post-intervention assessments were considered to have completed the study protocol. Participants who completed fewer than 21 training sessions, voluntarily withdrew from the study, or failed to complete the post-intervention assessments were classified as non-completers, and the specific reasons for non-completion were documented. Throughout the study, participants were instructed to maintain their habitual level of daily physical activity and to refrain from additional structured resistance training or other high-intensity exercise to minimize potential confounding from activities outside the study protocol.

### 2.5. Outcome Assessments

To ensure the reliability and reproducibility of the measurements, all outcome assessments were performed both before and after the intervention. Participants received standardized instructions regarding the testing procedures, precautions, and potential risks before each assessment. During the 48 h preceding testing, participants were instructed to abstain from alcohol, avoid medications that could affect exercise performance or endocrine function, refrain from using any nutritional supplements not specified in the study protocol, and avoid strenuous physical activity. Whenever possible, assessments were conducted at the same time of day and under standardized laboratory conditions to minimize potential influences of circadian variation, dietary status, fatigue, and environmental factors.

#### 2.5.1. Dietary Assessment and Control

Except on testing days, when standardized meals were provided by the research team, participants were instructed to maintain their habitual dietary and lifestyle patterns throughout the intervention. Dietary intake was recorded using the Boohee mobile nutrition analysis application (Boohee Information Technology Co., Ltd., Shanghai, China), which has previously been used as a dietary monitoring tool for quantifying food intake [22].

During the pre-screening phase, participants completed a 3-day dietary record comprising two weekdays and one weekend day. These records were used to estimate baseline total energy intake and macronutrient intake, including carbohydrate, fat, and protein consumption from meals and snacks. Throughout the 8-week intervention, participants recorded their dietary intake on three days each week, including two weekdays and one weekend day. Complete dietary records and corresponding photographs of consumed foods were submitted to the research team, and daily total energy and macronutrient intakes were subsequently quantified.

#### 2.5.2. Body Composition

Body composition was assessed using dual-energy X-ray absorptiometry (DXA; GE Lunar iDXA, Madison, WI, USA). The DXA system was calibrated before each measurement session according to the manufacturer’s procedures. The GE Lunar iDXA has demonstrated high precision for whole-body and regional body-composition measurements in previous test–retest studies [23]. Measurements were performed at 08:00. Participants had refrained from exercise for at least 8 h since their most recent training session and arrived in a fasted state. To minimize potential measurement variability, participants were also instructed to avoid caffeinated beverages before testing. Assessments were conducted in a quiet, temperature-controlled environment. The DXA system was calibrated before use, and participants wore light clothing and removed all metal objects.

Before scanning, each participant’s height, body mass, and age were entered into the system. Participants were then positioned supine in the center of the scanning table, with the body aligned along the midline of the scanner. During the scan, they were instructed to remain still with their eyes closed, palms facing downward, arms relaxed alongside the body, and feet together.

#### 2.5.3. Muscle Circumference Measurements

Muscle circumference was assessed using a flexible measuring tape at the upper arm, thigh, chest, and hip. All measurements were performed by the same investigator using identical anatomical landmarks, body positions, and measurement procedures before and after the intervention to ensure consistency.

Participants stood in a relaxed position with their feet shoulder-width apart and were instructed to avoid voluntary contraction of the muscles being measured. Upper-arm circumference was measured at the midpoint between the acromion and the elbow crease, with the tape positioned horizontally around the arm. Thigh circumference was measured at a standardized site located two-thirds of the distance from the anterior superior iliac spine to the superior border of the patella, with the same landmark used at both time points. Chest circumference was measured horizontally around the widest portion of the thorax and recorded at the end of a normal expiration. Hip circumference was measured horizontally at the point of maximal gluteal prominence.

During each measurement, the tape was placed snugly against the skin without compressing the underlying soft tissue. Measurements were recorded to the nearest 0.1 cm. Each site was measured three times; if the difference between repeated measurements exceeded 0.5 cm, the measurement was repeated. The mean of the three measurements was used for subsequent analysis. Intra-rater reliability was assessed using the three repeated measurements obtained at baseline. ICCs were calculated using a two-way mixed-effects model with absolute agreement for average measures, and coefficients of variation (CVs) were calculated to quantify repeated-measurement variability. Circumference measurements demonstrated excellent intra-rater reliability, with ICCs ranging from 0.995 to 0.998 and CVs ranging from 0.63% to 0.79%.

#### 2.5.4. Muscle Thickness Measurements

Muscle thickness was assessed using a GE Vivid 7 color Doppler ultrasound system (GE Vivid 7; GE Medical Systems, Milwaukee, WI, USA). Measurements were obtained for the quadriceps femoris, biceps brachii, and pectoralis major. Participants were instructed to avoid strenuous exercise before testing to minimize the potential influence of exercise-induced hyperemia or edema on muscle thickness measurements. All ultrasound assessments were performed by the same experienced investigator using identical device settings, participant positioning, and anatomical landmarks at baseline and post-intervention.

Participants were examined in a relaxed supine position, with positioning adjusted according to the muscle being assessed. For the quadriceps femoris, participants lay supine with both legs extended and relaxed, and a standardized measurement site was marked on the anterior aspect of the dominant thigh at 2/3 of the distance between the anterior superior iliac spine and the superior border of the patella. For the biceps brachii, the arm was kept relaxed, and the measurement site was located on the anterior aspect of the right upper arm at the midpoint between the acromion and the elbow crease. For the pectoralis major, participants remained supine with the upper limbs relaxed, and measurements were obtained at a fixed site corresponding to the intersection of the right midclavicular line and the fourth intercostal space. All measurement sites were marked and documented during the baseline assessment to ensure consistent positioning at follow-up.

A sufficient amount of ultrasound gel was applied to the skin, and the transducer was positioned perpendicular to the skin surface with minimal pressure to avoid compression of the underlying tissue. The probe angle was adjusted until the boundaries of the target muscle, and the superficial and deep fasciae were clearly visualized. Three clear images were acquired at each measurement site, and muscle thickness was determined using the ultrasound system software. The mean of the three measurements was used for subsequent analysis. Images were reacquired if the muscle boundaries were not clearly identifiable or if visible muscle contraction occurred during measurement. The intra-rater reliability of ultrasound-derived muscle thickness measurements was high, with ICCs ranging from 0.937 to 0.997 and CVs ranging from 1.48% to 7.63% across the measured sites.

#### 2.5.5. Muscular Strength Testing

Muscular strength was assessed using standardized procedures based on recommendations from the National Strength and Conditioning Association. One-repetition maximum (1RM) and eight-repetition maximum (8RM) were determined for the squat, deadlift, and bench press. Before formal testing, all participants completed familiarization sessions during which the research staff provided standardized instruction and demonstrations of exercise technique, including starting position, range of motion, lifting technique, breathing pattern, and safety procedures. At least 48 h separated the familiarization sessions from formal strength testing to minimize potential learning and fatigue effects.

Before testing, participants completed a standardized warm-up consisting of 5–10 min of low-intensity aerobic exercise, dynamic stretching, and exercise-specific warm-up sets. The 1RM test was performed using a progressive loading protocol. Participants first completed 8–10 repetitions at approximately 50% of their estimated 1RM, followed by 2 min of rest and 3–5 repetitions at approximately 70% of estimated 1RM. The load was then progressively increased until the participant could complete one repetition with proper technique and full range of motion. Rest intervals of 3–5 min were provided between attempts, and 1RM was generally established within 3–5 maximal attempts for each exercise. If a repetition was completed successfully with correct technique, the load was increased after adequate recovery. If the attempt was unsuccessful, the highest successfully completed load was recorded as the 1RM.

The 8RM test was used to assess strength performance under a moderate-repetition load. The 8RM was defined as the greatest load that could be lifted for eight consecutive repetitions with correct technique and full range of motion, but not for a ninth repetition. The 8RM assessment was conducted at least 48 h apart from the 1RM test. Following a standardized exercise-specific warm-up, the initial load was estimated from the participant’s 1RM or familiarization performance. If more than eight repetitions could be completed, the load was increased for the subsequent attempt; if fewer than eight repetitions were completed, the load was reduced. Rest intervals of 3–5 min were provided between attempts, with no more than 3–5 formal attempts per exercise. The highest load successfully completed for eight repetitions was recorded as the 8RM.

For the squat, participants were required to maintain trunk stability and full-foot contact throughout the movement, while avoiding excessive forward trunk lean, knee valgus, or the use of rebound momentum. During the bench press, the scapulae were kept stable, and the buttocks remained in contact with the bench; the barbell was lowered under control to the chest and then pressed until the elbows were fully extended. For the deadlift, participants lifted the barbell from the floor to full hip and knee extension while maintaining a stable spinal position. Attempts were considered invalid if clear compensatory movements, insufficient range of motion, or loss of proper technique occurred.

All strength assessments were conducted by the same research team, with spotters present to ensure participant safety. Standardized verbal encouragement was provided during each test, and the highest valid load was recorded for each exercise. Testing was terminated immediately if a participant experienced joint pain, marked discomfort, or any other safety concern.

#### 2.5.6. Blood Biomarker Analysis

Blood biomarkers were assessed using a UniCel DxI 800 Access Immunoassay System (Beckman Coulter, Inc., Brea, CA, USA). Serum testosterone and cortisol concentrations were measured, and the testosterone-to-cortisol (T/C) ratio was subsequently calculated. To minimize the influence of circadian variation and acute exercise-induced stress on hormone concentrations, all blood samples were collected in the morning within the same time window. Participants were required to fast for approximately 12 h before blood collection and to avoid strenuous exercise, alcohol consumption, and the use of medications or supplements that could affect endocrine function for 48 h before testing.

Venous blood samples were collected from the antecubital vein by qualified medical personnel. After collection, samples were allowed to clot at room temperature and were then centrifuged according to the assay requirements. Serum was separated and stored under refrigerated conditions until analysis. Serum testosterone and cortisol concentrations were determined using the automated chemiluminescence immunoassay system, with all samples analyzed in accordance with the manufacturer’s instructions and standard operating procedures.

### 2.6. Statistical Analysis

Statistical analyses were performed using SPSS Statistics version 26.0 (IBM Corp., Armonk, NY, USA). Continuous variables are presented as mean ± standard deviation (SD) or standard error (SE), as appropriate. The assumptions underlying the parametric analyses were assessed before statistical testing. Normality was evaluated using the Shapiro–Wilk test together with visual inspection of Q–Q plots, and homogeneity of variance was assessed using Levene’s test. As the within-subject factor included only two time points (pre- and post-intervention), the assumption of sphericity was inherently satisfied.

Baseline characteristics and between-group differences were evaluated using one-way analysis of variance (ANOVA). Changes over time and differences in responses among groups were examined using a two-way mixed-design ANOVA, with time as the within-subject factor and group as the between-subject factor. Main effects of time and group, as well as the time × group interaction, were assessed. When a significant time × group interaction was identified, simple-effects analyses were performed, with Bonferroni adjustment applied to pairwise between-group comparisons within each outcome. All statistical tests were two-sided, and statistical significance was set at *p* < 0.05. Effect sizes were additionally reported to facilitate interpretation of the magnitude of the observed effects. For the mixed-design ANOVA, effect sizes were expressed as partial eta squared (ηp^2^). For principal pairwise between-group comparisons, standardized effect sizes were calculated as Hedges’ g and are presented with 95% confidence intervals. Statistical power was addressed a priori during sample-size estimation using G*Power, with a target power of 80% (1 − β = 0.80) at a two-sided α level of 0.05.

Intra-rater reliability for circumference and ultrasound-derived muscle thickness measurements was assessed using the three repeated measurements obtained at baseline. Intraclass correlation coefficients (ICCs) were calculated using a two-way mixed-effects model with absolute agreement for average measures. Coefficients of variation (CVs) were additionally calculated to quantify within-subject measurement variability.

## 3. Results

The Shapiro–Wilk tests indicated no significant deviations from normality across the analyzed outcomes (*p* = 0.11–0.94), while Levene’s tests indicated that the assumption of homogeneity of variance was satisfied (*p* = 0.24–0.88). These results supported the use of the planned parametric analyses.

### 3.1. Participant Adherence

A total of 36 participants were enrolled in the study, of whom 33 completed the full intervention and post-intervention assessments, corresponding to an overall completion rate of 91.7%. All study completers met the prespecified adherence criteria for both training and supplementation. Training adherence rates were 95.8%, 96.2%, and 97.2% in the PLA, LMW-CHO+PRO, and HMW-CHO+PRO groups, respectively, with no significant between-group difference (*p* = 0.684). Supplement adherence rates were 92.5%, 93.2%, and 92.8%, respectively, and did not differ significantly among the three groups (*p* = 0.815).

### 3.2. Nutritional Intake

As shown in Table 5, no significant between-group differences were observed at baseline in mean daily energy, protein, carbohydrate, or fat intake (all *p* > 0.05).

During the intervention, mean daily intake across all participants was 2076.5 ± 272.4 kcal for energy, 116.6 ± 24.2 g for protein, 225.8 ± 42.7 g for carbohydrate, and 78.2 ± 15.6 g for fat. No significant between-group differences were observed in energy (*p* = 0.361), absolute protein (*p* = 0.151), relative protein (*p* = 0.145), carbohydrate (*p* = 0.317), or fat intake (*p* = 0.908). Although relative protein intake was numerically higher in the two carbohydrate–protein groups during the intervention, no additional dietary changes were prescribed, and participants were instructed to maintain their habitual diet and lifestyle apart from the assigned supplementation.

### 3.3. Effects of the Intervention on Changes in Body Composition

Following the 8-week intervention combining nutritional supplementation with resistance training, body mass and total muscle mass increased significantly from baseline in all three groups (all Bonferroni-adjusted *p* < 0.001). Two-way mixed-design ANOVA revealed significant time × group interactions for both body mass [*F*(2, 30) = 8.220, *p* = 0.001, ηp^2^ = 0.354] and total muscle mass [*F*(2, 30) = 16.322, *p* < 0.001, ηp^2^ = 0.521], whereas the time × group interaction for fat mass was not significant [*F*(2, 30) = 1.901, *p* = 0.167, ηp^2^ = 0.112]. Body composition data are presented in Table 6.

Further pairwise comparisons showed that increases in body mass and total muscle mass were significantly greater in both the LMW-CHO+PRO and HMW-CHO+PRO groups than in the PLA group (*p* < 0.05), with greater increases also observed in the HMW-CHO+PRO group than in the LMW-CHO+PRO group (*p* < 0.05). For body mass, Hedges’ g values were 1.18 (95% CI: 0.25–2.12), 2.52 (95% CI: 1.37–3.67), and 0.92 (95% CI: 0.06–1.79) for LMW-CHO+PRO versus PLA, HMW-CHO+PRO versus PLA, and HMW-CHO+PRO versus LMW-CHO+PRO, respectively. The corresponding values for total muscle mass were 1.27 (95% CI: 0.33–2.22), 2.77 (95% CI: 1.57–3.97), and 1.28 (95% CI: 0.37–2.18), respectively. For regional muscle mass, significant between-group differences in pre-to-post changes were observed for upper-limb muscle mass [*F*(2, 30) = 4.040, *p* = 0.028, ηp^2^ = 0.212], thigh muscle mass [*F*(2, 30) = 6.204, *p* = 0.006, ηp^2^ = 0.293], and trunk muscle mass [*F*(2, 30) = 4.985, *p* = 0.014, ηp^2^ = 0.249]. Overall, these findings indicate that, alongside systematic resistance training, both LMW-CHO+PRO and HMW-CHO+PRO supplementation were associated with greater increases in body mass and total muscle mass, with the increase in total muscle mass being significantly greater in the HMW-CHO+PRO group than in the LMW-CHO+PRO group.

### 3.4. Effects of the Intervention on Changes in Muscle Circumference

**Figure 2:**
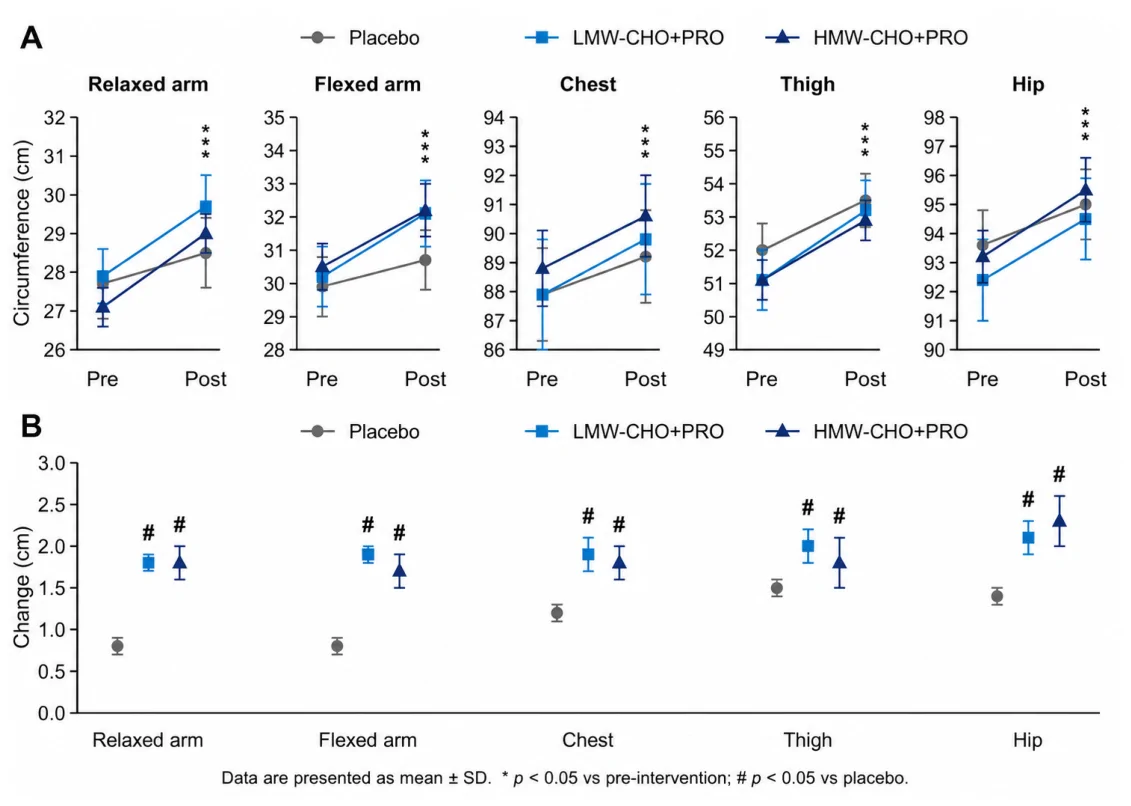
Changes in muscle circumference before and after the intervention. (**A**) Pre- and post-intervention measurements of relaxed arm, flexed arm, chest, thigh, and hip circumference. (**B**) Changes from baseline. PLA, placebo; LMW-CHO+PRO, low-molecular-weight carbohydrate plus protein; HMW-CHO+PRO, high-molecular-weight carbohydrate plus protein.

After the 8-week intervention, relaxed arm circumference, flexed arm circumference, chest circumference, thigh circumference, and hip circumference increased significantly from baseline in all three groups (*p* < 0.05). Compared with the PLA group, both the LMW-CHO+PRO and HMW-CHO+PRO groups showed significantly greater increases across all circumference measures (*p* < 0.05). No significant differences were observed between the two carbohydrate–protein formulation groups (*p* > 0.05). These findings indicate that both supplementation strategies were associated with greater increases in body circumference during resistance training, with no clear difference between the two formulations (Figure 2).

### 3.5. Effects of the Intervention on Changes in Muscle Thickness

**Figure 3:**
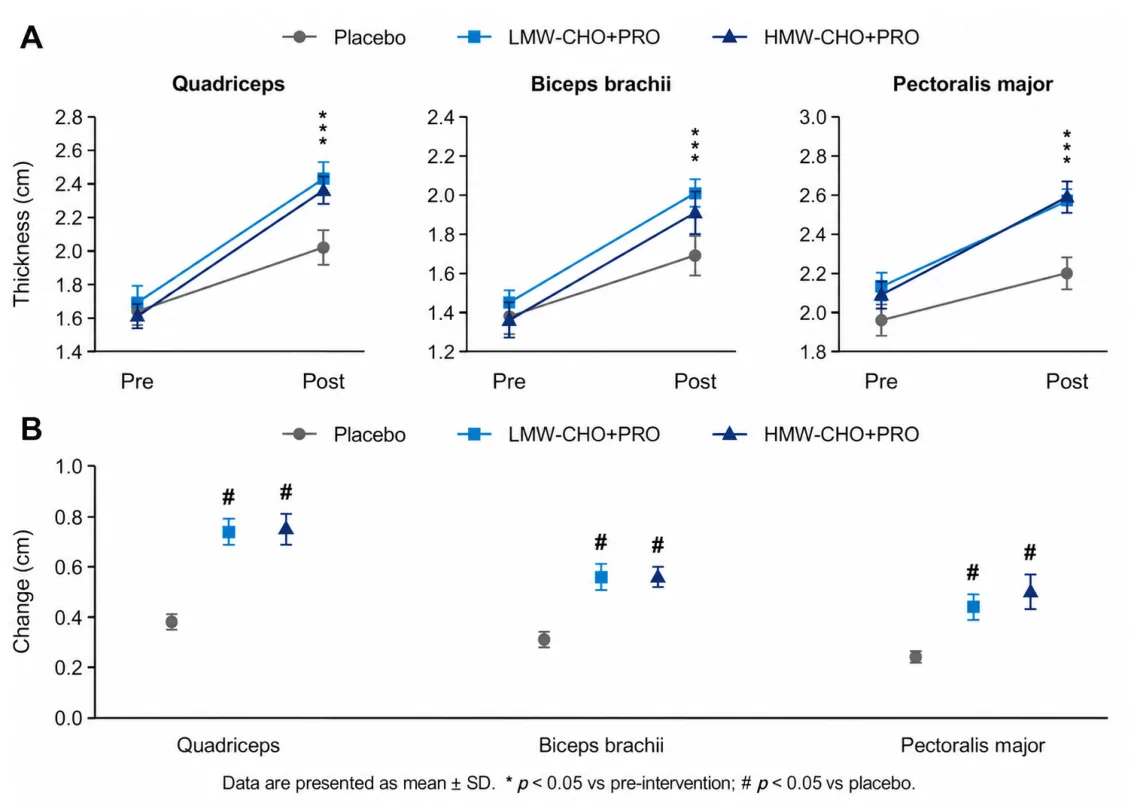
Changes in muscle thickness before and after the intervention. (**A**) Pre- and post-intervention measurements of the quadriceps, biceps brachii, and pectoralis major. (**B**) Changes from baseline. PLA, placebo; LMW-CHO+PRO, low-molecular-weight carbohydrate plus protein; HMW-CHO+PRO, high-molecular-weight carbohydrate plus protein.

Muscle thickness increased significantly from baseline in the quadriceps femoris, biceps brachii, and pectoralis major in all three groups (*p* < 0.05). The increases in muscle thickness at all measured sites were significantly greater in both the LMW-CHO+PRO and HMW-CHO+PRO groups than in the placebo group (*p* < 0.05), whereas no significant differences were observed between the two supplementation groups (*p* > 0.05) (Figure 3).

### 3.6. Effects of the Intervention on Changes in Muscular Strength

**Figure 4:**
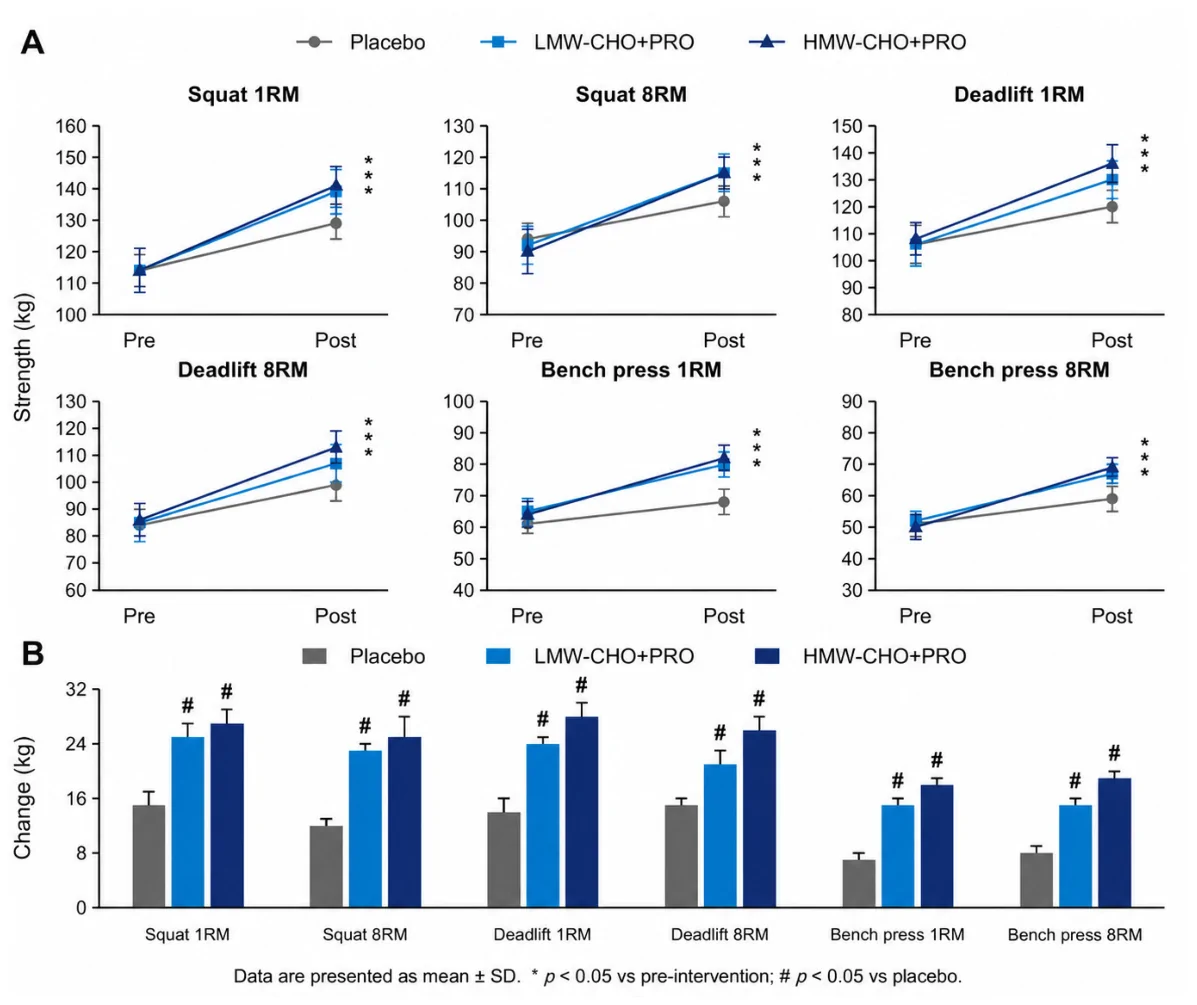
Changes in muscular strength before and after the intervention. (**A**) Pre- and post-intervention squat, deadlift, and bench press performance. (**B**) Changes from baseline. 1RM, one-repetition maximum; 8RM, eight-repetition maximum. PLA, placebo; LMW-CHO+PRO, low-molecular-weight carbohydrate plus protein; HMW-CHO+PRO, high-molecular-weight carbohydrate plus protein.

After the 8-week intervention, 1RM and 8RM performance in the squat, deadlift, and bench press increased significantly from baseline in all three groups (*p* < 0.05). Compared with the placebo group, both the LMW-CHO+PRO and HMW-CHO+PRO groups showed significantly greater improvements across all strength outcomes (*p* < 0.05), whereas no significant differences were observed between the two supplementation groups (*p* > 0.05) (Figure 4).

### 3.7. Effects of the Intervention on Changes in Blood Biomarkers

Serum testosterone concentrations increased significantly from baseline in both the LMW-CHO+PRO and HMW-CHO+PRO groups (*p* < 0.05), and the magnitude of these increases was significantly greater than that observed in the placebo group (*p* < 0.05). No significant change in testosterone was observed in the placebo group (*p* > 0.05).

Similarly, serum cortisol concentrations decreased significantly from baseline in both supplementation groups (*p* < 0.05), with significantly greater reductions than in the placebo group (*p* < 0.05). Cortisol concentrations did not change significantly in the placebo group (*p* > 0.05).

**Figure 5:**
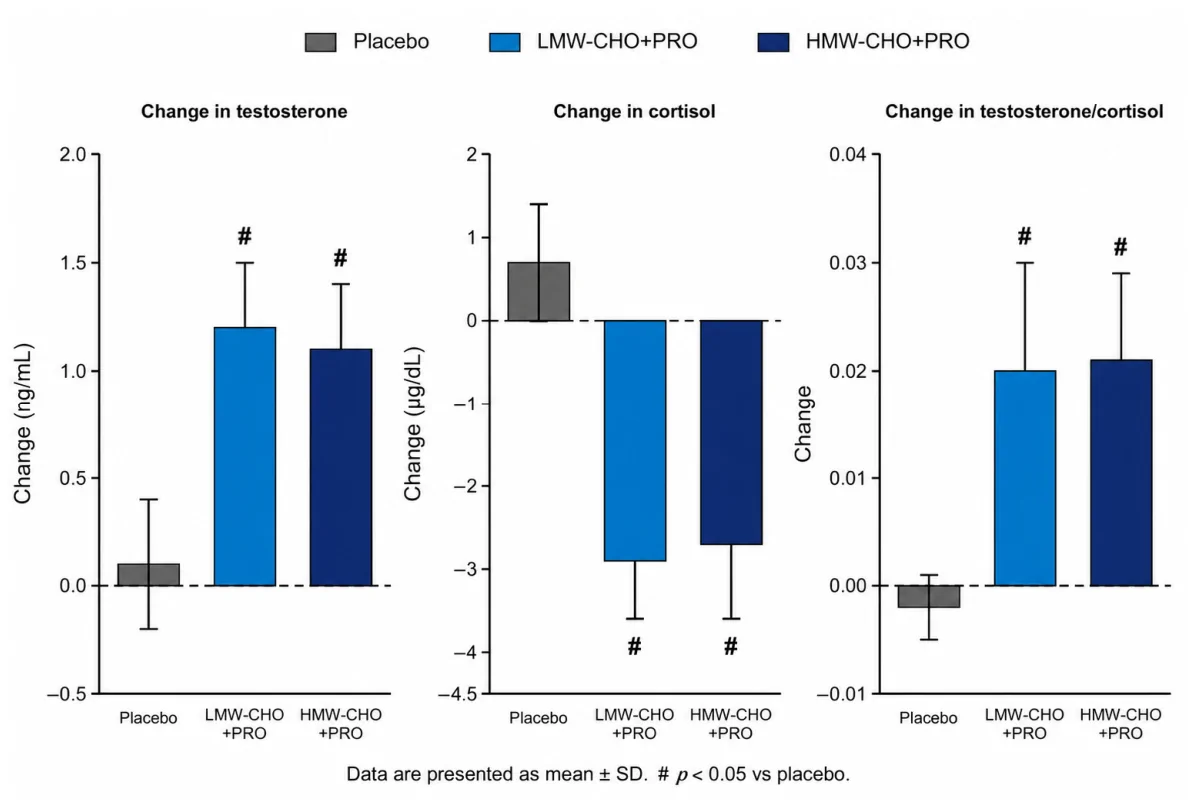
Changes in blood biomarkers before and after the intervention. From left to right: testosterone, cortisol, and testosterone-to-cortisol ratio. PLA, placebo; LMW-CHO+PRO, low-molecular-weight carbohydrate plus protein; HMW-CHO+PRO, high-molecular-weight carbohydrate plus protein.

The testosterone-to-cortisol (T/C) ratio also increased significantly after the intervention in both the LMW-CHO+PRO and HMW-CHO+PRO groups (*p* < 0.05), and these increases were significantly greater than those in the placebo group (*p* < 0.05). No significant change in the T/C ratio was observed in the placebo group (Figure 5).

## 4. Discussion

This study compared the effects of different carbohydrate–protein supplementation strategies on resistance training adaptations in healthy young men. The main findings showed that, following 8 weeks of structured resistance training, body mass, skeletal muscle mass, muscle circumference, muscle thickness, and muscular strength increased significantly from baseline in all three groups. Beyond these training-induced adaptations, the LMW-CHO+PRO and HMW-CHO+PRO groups exhibited greater improvements than the placebo group across most measures of muscular adaptation, suggesting that combined nutritional supplementation may provide additional support for resistance training adaptations. Differences between the two active formulations were primarily observed for body mass and total muscle mass, with a greater increase in the HMW-CHO+PRO group than in the LMW-CHO+PRO group; however, this difference was not consistently reflected in regional muscle morphology, strength outcomes, or blood biomarkers. Therefore, this isolated finding should be interpreted cautiously and regarded as exploratory and hypothesis-generating rather than as confirmatory evidence of greater muscle hypertrophy.

Resistance training can promote skeletal muscle protein synthesis and structural remodeling through stimuli such as mechanical tension, metabolic stress, and muscle fiber microdamage, thereby contributing to increases in muscle mass [24]. Previous studies have shown that prolonged resistance training effectively induces muscle hypertrophy, with training volume exhibiting a dose–response relationship with hypertrophic adaptation [25]. Training frequency and load intensity may further modulate these responses by influencing total training volume, the distribution of training stimuli, and accumulated fatigue [26]. In the present study, body mass and muscle mass increased across all three groups, whereas fat mass remained relatively stable, suggesting that the gain in body mass was accompanied primarily by an increase in lean tissue. Strength also improved in all groups, likely reflecting a combination of muscle hypertrophy, enhanced neuromuscular coordination, and greater movement proficiency. In young men with relatively limited prior resistance-training experience, neural adaptations may make a particularly important contribution during the early stages of training [27,28,29].

When interpreting the differences between the combined formulations and the control condition, the nature of the control treatment should be carefully considered. Because the PLA group received maltodextrin, which provided both carbohydrate and energy, it served as a carbohydrate control rather than a physiologically inert placebo. Accordingly, the present study should be interpreted primarily as a comparison of protein-containing carbohydrate–protein formulations with a carbohydrate-only maltodextrin control, rather than as a comparison between nutritional supplementation and a non-supplemented condition. Accordingly, the advantages observed for the LMW-CHO+PRO and HMW-CHO+PRO groups relative to PLA should be interpreted as differences between protein-containing mixed nutritional formulations and maltodextrin-based carbohydrate supplementation. Protein provides the essential amino acids required for post-exercise muscle repair and remodeling and is an important nutritional determinant of gains in fat-free mass and muscular strength [11,30]. In individuals who train regularly, adequate daily protein intake is fundamental to supporting post-exercise recovery, tissue remodeling, and long-term adaptation [31]. Carbohydrate, by contrast, primarily supports energy and blood glucose availability during exercise, helps limit muscle glycogen depletion, and facilitates post-exercise glycogen restoration, thereby supporting subsequent training and recovery. Co-ingestion of carbohydrate and protein may also create a more favorable nutritional environment for recovery and lean-tissue accretion [9,32]. Previous studies have suggested that combined supplementation with carbohydrate and protein or essential amino acids during resistance training may contribute to improvements in fat-free mass and muscular adaptation [33]. Therefore, the differences observed between the two intervention groups and the PLA group in the present study are more appropriately interpreted as effects of the overall nutritional supplementation strategy rather than as being attributable to any single nutrient component.

Further comparison of the two mixed formulations showed that the increase in total muscle mass was greater in the HMW-CHO+PRO group than in the LMW-CHO+PRO group. The two products provided comparable amounts of protein and similar overall macronutrient content, with their main differences lying in carbohydrate sources and composition. The LMW-CHO+PRO formulation consisted primarily of maltodextrin, maltooligosaccharide powder, and glucose, whereas the HMW-CHO+PRO formulation contained isomaltulose, highly branched cyclic dextrin, Vitargo^®^ modified starch, crystalline fructose, and other carbohydrate sources. Previous studies have shown that different carbohydrate types vary in molecular structure, degree of polymerization, and absorption characteristics, resulting in differences in solution osmolality, gastric emptying, and glycemic and insulinemic responses, which may in turn influence post-exercise glycogen resynthesis and substrate restoration [15,34,35,36]. Some studies have reported that high-molecular-weight carbohydrates may accelerate muscle glycogen resynthesis during the early recovery period and help maintain movement velocity and power output during subsequent bouts of high-intensity exercise [37,38]. Piehl Aulin et al. found that, compared with an isoenergetic low-molecular-weight carbohydrate, a high-molecular-weight carbohydrate increased the rate of muscle glycogen synthesis during the first 2 h of recovery [37]. Oliver et al. further reported that ingestion of a high-molecular-weight carbohydrate improved velocity and power during repeated maximal resistance exercise following glycogen-depleting exercise [38]. These findings provide a physiological basis for the possibility that different carbohydrate formulations may elicit distinct responses. However, such studies have focused primarily on acute metabolic recovery or exercise performance, and whether these acute effects translate into long-term differences in muscle mass remains unclear. From a mechanistic perspective, carbohydrate structure, concentration, and solution osmolality may influence gastric emptying and intestinal absorption [39,40], with potential downstream effects on post-exercise carbohydrate utilization and muscle glycogen restoration [41,42].

Moreover, the HMW-CHO+PRO formulation used in the present study was not composed of a single high-molecular-weight carbohydrate. Rather, the two products differed simultaneously in carbohydrate sources, molecular characteristics, relative proportions, and estimated glycemic index (GI). The estimated GI values were approximately 80.2 and 66.7 for the LMW-CHO+PRO and HMW-CHO+PRO formulations, respectively. However, these values were theoretical estimates rather than direct measurements obtained in human participants and may also have been influenced by protein co-ingestion and product processing. Therefore, the greater increase in total muscle mass observed in the HMW-CHO+PRO group should be interpreted as an effect of the overall formulation and cannot be attributed specifically to carbohydrate molecular weight or differences in GI.

Although the HMW-CHO+PRO group showed a significantly greater increase in total skeletal muscle mass than the LMW-CHO+PRO group, no significant between-group differences were observed in muscle circumference or muscle thickness. This discrepancy may partly reflect differences in the biological characteristics captured by the respective measurement methods and their sensitivity to change. DXA-derived estimates of muscle mass and lean mass reflect whole-body or regional tissue changes and may be influenced by variations in muscle glycogen stores and intracellular fluid content [43,44]. By contrast, ultrasound-derived muscle thickness and circumference measurements primarily reflect morphological changes at specific anatomical sites and may be less sensitive to subtle short-term between-group differences. Therefore, the observed difference in total muscle mass alone is insufficient to conclude that one formulation produced a greater hypertrophic response. From a physiological perspective, protein and carbohydrate may have complementary roles in supporting recovery from resistance exercise, with protein providing amino acids for muscle remodeling and carbohydrate promoting glycogen restoration and training quality. However, potential acute differences in carbohydrate handling, such as gastric emptying or glycogen resynthesis, may not necessarily translate into greater long-term hypertrophy or strength. This may partly explain why the DXA-derived difference in total muscle mass between the two formulations was not consistently reflected in muscle thickness, circumference, or strength.

No significant differences in strength adaptations were observed between the two mixed formulations, a finding that was broadly consistent with the results for regional muscle morphology and further suggests that the between-group difference in total muscle mass had not translated into a detectable functional advantage. With respect to blood biomarkers, both the LMW-CHO+PRO and HMW-CHO+PRO groups showed more favorable changes in testosterone, cortisol, and the testosterone-to-cortisol (T/C) ratio than the PLA group, whereas no clear differences were observed between the two active formulations. Testosterone is an important endocrine factor involved in skeletal muscle anabolism and adaptation to resistance training, whereas cortisol primarily reflects physiological responses related to training stress and energy mobilization. Sustained or excessive elevations in cortisol may suppress muscle protein synthesis, promote protein breakdown, and have been associated with reductions in muscle mass and strength as well as impaired recovery [45]. The T/C ratio is commonly used as an auxiliary marker of training-related endocrine responses and anabolic–catabolic status [46]. However, resting circulating hormone concentrations are influenced by multiple factors, including sleep, energy intake, psychological state, and recent training load. Moreover, within the normal physiological range in healthy individuals, resting testosterone and cortisol concentrations, as well as transient exercise-induced hormonal responses, do not show a consistent relationship with long-term muscle hypertrophy or strength gains [47,48,49]. These secondary biomarkers should therefore be regarded as supportive indicators of endocrine status during training rather than as evidence of a direct mechanism underlying changes in muscle mass or strength.

This study employed a randomized, double-blind design, with resistance training performed under standardized supervision. Dietary intake, training adherence, and supplement adherence were monitored throughout the intervention. In addition, intervention effects were evaluated using a combination of body composition, regional muscle morphology, strength performance, and blood biomarkers, allowing training adaptations to be assessed across multiple domains. However, several limitations should be acknowledged. Several limitations should be acknowledged. First, the modest sample size (10–12 participants per group) may have limited the precision and stability of the between-group estimates. In addition, multiple outcomes were examined in this relatively small sample, which may increase the possibility of chance findings. Accordingly, statistically significant findings that were not consistently supported across related morphological and functional outcomes should be regarded as exploratory and interpreted cautiously. Second, dietary intake was monitored using 3-day dietary records each week rather than being fully controlled, and day-to-day variation on non-recording days therefore cannot be excluded. Third, the 8-week intervention included only healthy young men with limited prior resistance-training experience, which limits the generalizability of the findings to longer training periods and other populations. Fourth, because the intervention products were mixed formulations that differed in multiple compositional characteristics, the independent effects of any single carbohydrate source or carbohydrate molecular weight could not be isolated. Finally, the study did not directly assess muscle glycogen, postprandial glucose and insulin responses, gastric emptying, or muscle protein synthesis, and therefore cannot establish the specific physiological mechanisms underlying the observed effects. In particular, the between-formulation difference in DXA-derived total muscle mass should be interpreted cautiously, as DXA estimates may be influenced by glycogen-associated fluid shifts and this finding was not consistently corroborated by regional muscle thickness, circumference, or strength outcomes. Overall, when combined with 8 weeks of structured resistance training, both carbohydrate–protein formulations appeared to provide additional support for gains in muscle mass, regional muscle morphology, and strength in healthy young men compared with the maltodextrin-based carbohydrate control. The HMW-CHO+PRO group showed greater increases in body mass and total muscle mass; however, these differences were not consistently observed across other outcomes and therefore do not provide sufficient evidence for an overall advantage of this formulation. From a practical perspective, both formulations may be considered viable nutritional supplementation options during resistance training, but the present findings do not justify preferentially recommending one over the other. Future studies should include larger samples and longer intervention periods and, under conditions in which energy, protein, and carbohydrate doses are tightly matched, incorporate direct measures of glycogen metabolism, postprandial metabolic responses, and muscle protein synthesis to clarify the long-term effects of different carbohydrate–protein formulations.

## 5. Conclusions

This study demonstrated that 8 weeks of structured resistance training improved muscle mass, muscle morphology, and muscular strength in healthy young men. Compared with the maltodextrin-based carbohydrate control group, the two protein-containing carbohydrate–protein formulations were associated with greater improvements in several measures of training adaptation. However, because the active formulations also provided additional protein, the present design cannot determine whether these differences were attributable to protein, carbohydrate characteristics, or the overall formulations. Between the two formulations, the HMW-CHO+PRO group showed greater increases in body mass and total muscle mass than the LMW-CHO+PRO group, whereas no consistent between-group differences were observed in regional muscle circumference, muscle thickness, muscular strength, or blood biomarkers. Therefore, under conditions of relatively adequate protein intake, different carbohydrate–protein formulations may differentially influence certain aspects of resistance training adaptation. However, the present findings do not establish the superiority of either formulation, and the observed difference in total muscle mass cannot be attributed specifically to carbohydrate molecular weight because the two products differed in multiple carbohydrate sources and other compositional characteristics.

## Figures and Tables

**Table 1:** Baseline characteristics of the participants.

Variable	All Participants (*N* = 33)	PLA (*n* = 10)	LMW-CHO+PRO (*n* = 11)	HMW-CHO+PRO (*n* = 12)
Age (years)	22 ± 3	21 ± 2	22 ± 3	23 ± 3
Height (cm)	177.4 ± 6.1	176.7 ± 4.5	179.1 ± 7.3	176.2 ± 6.4
Body mass (kg)	68.6 ± 2.3	68.4 ± 1.9	68.4 ± 2.8	67.5 ± 2.2
BMI (kg/m^2^)	21.8 ± 1.6	21.9 ± 1.7	21.4 ± 1.6	22.0 ± 1.6

Note: LMW-CHO+PRO, low-molecular-weight carbohydrate plus protein supplementation group; HMW-CHO+PRO, high-molecular-weight carbohydrate plus protein supplementation group; PLA, placebo group. BMI, body mass index.

**Table 2:** Nutritional composition of the supplements.

Per 50 g Serving	LMW-CHO+PRO	HMW-CHO+PRO
Energy (kJ)	777	791.5
Protein (g)	12.5	12.5
Fat (g)	0.25	0.25
Carbohydrate (g)	32.3	33.15
Dietary fiber (g)	0.75	0.75
Sodium (mg)	38	10

**Table 3:** Main ingredients of the supplements.

LMW-CHO+PRO	HMW-CHO+PRO
Whey protein concentrate, whey protein isolate, milk protein, maltodextrin, maltooligosaccharide powder, and glucose.	Whey protein concentrate, whey protein isolate, milk protein, maltodextrin, isomaltulose, maltooligosaccharide powder, highly branched cyclic dextrin, Vitargo^®^ modified starch, and crystalline fructose.

**Table 4:** Resistance Training Program.

Muscle Group	Weeks 1–4 (Adaptation Phase)	Weeks 5–8 (Intensification Phase)
Standardized warm-up: 10 min		
Legs	Squat, deadlift (75% 1RM, 8 repetitions × 3 sets)	Squat, deadlift (85% 1RM, 5 repetitions × 3 sets)
Chest	Bench press (75% 1RM, 8 repetitions × 4 sets)	Bench press (85% 1RM, 5 repetitions × 3 sets)
Back	Seated row, lat pulldown (8RM, 3 sets)	Seated row, lat pulldown (5RM, 3 sets)
Core	Back extension, hanging leg raise (8–12 repetitions × 3 sets)	Back extension, hanging leg raise (8–12 repetitions × 3 sets)
Cool-down: 10 min Training volume = load (kg) × repetitions × sets × frequency		

**Table 5:** Dietary Intake at Baseline and During the Intervention.

		All Subjects	PLA	LMW-CHO+PRO	HMW-CHO+PRO	*F*	*p*
Baseline daily	Energy (kcal/d)	1885.8 ± 438.5	1773 ± 341	1987 ± 428.3	1887 ± 525	0.609	0.551
	Protein (g/d)	89.3 ± 23.3	89 ± 28.6	87 ± 20	91.7 ± 23.1	0.112	0.895
	Protein (g/kg/day)	1.21 ± 0.32	1.20 ± 0.38	1.18 ± 0.27	1.24 ± 0.31	0.125	0.883
	Carbohydrate (g/d)	188.4 ± 57.1	182 ± 60	197.8 ± 53	185 ± 62	0.222	0.802
	Fat (g/d)	66.9 ± 24.7	70 ± 28	63 ± 25	68 ± 23.2	0.217	0.806
Intervention period	Energy (kcal/d)	2076.5 ± 272.4	1993 ± 284	2068 ± 251	2154 ± 276	1.052	0.361
	Protein (g/d)	116.6 ± 24.2	105 ± 23	118 ± 22	125 ± 25	2.016	0.151
	Protein (g/kg/day)	1.58 ± 0.33	1.42 ± 0.31	1.60 ± 0.30	1.69 ± 0.34	2.053	0.145
	Carbohydrate (g/d)	225.8 ± 42.7	210 ± 40	227 ± 45	238 ± 42	1.193	0.317
	Fat (g/d)	78.2 ± 15.6	80 ± 15	78 ± 16	77 ± 17	0.097	0.908

Note: Data are presented as mean ± standard deviation (mean ± SD). At baseline, mean daily total intake was calculated as the sum of nutrients consumed at breakfast, lunch, dinner, and snacks. During the intervention, mean daily total energy and macronutrient intakes, including protein, fat, and carbohydrate (CHO), were calculated by summing intake from breakfast, lunch, dinner, snacks, and the study-provided nutritional supplement or placebo. PLA, placebo; LMW-CHO+PRO, low-molecular-weight carbohydrate plus protein; HMW-CHO+PRO, high-molecular-weight carbohydrate plus protein.

**Table 6:** Changes in Body Composition Before and After the Intervention (kg).

Variable/Time Point		PLA	LMW-CHO+PRO	HMW-CHO+PRO
Body Mass	Pre	68.4 ± 1.9	68.4 ± 2.8	67.5 ± 2.2
	Post	69.8 ± 2.0 *	70.5 ± 2.8 *	70.1 ± 2.1 *
	Change	1.3 ± 0.2	2.1 ± 0.2 #	2.6± 0.1 #▲
Muscle Mass	Pre	54.7 ± 1.9	54.6 ± 1.9	53.8 ± 1.9
	Post	55.5 ± 1.9 *	56.1 ± 2.0 *	56.2 ± 1.8 *
	Change	0.8 ± 0.1	1.5 ± 0.2 #	2.4 ± 0.2 #▲
Fat Mass	Pre	10.9 ± 1.3	11.0 ± 1.5	10.9 ± 0.9
	Post	11.4 ± 1.4	11.3 ± 1.5	10.9 ± 1.0
	Change	0.5 ± 0.1	0.3 ± 0.1	0.1 ± 0.2
Upper-Limb Muscle Mass	Pre	6.4 ± 0.3	6.8 ± 0.4	6.5 ± 0.4
	Post	6.6 ± 0.3	7.3 ± 0.4 *	7.0 ± 0.3 *
	Change	0.2 ± 0.1	0.5 ± 0.2 #	0.5 ± 0.1 #
Thigh Muscle Mass	Pre	19.2 ± 0.7	19.2 ± 0.7	19.1 ± 0.8
	Post	19.3 ± 0.6	19.7 ± 0.7 *	19.7 ± 0.8 *
	Change	0.1 ± 0.1	0.6 ± 0.1 #	0.6 ± 0.1 #
Trunk Muscle Mass	Pre	25.7 ± 0.9	25.8 ± 1.0	25.5 ± 0.9
	Post	26.1 ± 1.0	26.5 ± 1.0 *	26.6 ± 0.8 *
	Change	0.4 ± 0.2	0.7 ± 0.1 #	1.1 ± 0.1 #

Note: Data are presented as mean ± standard error (mean ± SE). * indicates a significant pre- to post-intervention difference (*p* < 0.05); # indicates a significant difference in the change from baseline compared with the placebo group (*p* < 0.05); ▲ indicates a significant difference in the change from baseline between the LMW-CHO+PRO and HMW-CHO+PRO groups (*p* < 0.05). PLA, placebo; LMW-CHO+PRO, low-molecular-weight carbohydrate plus protein; HMW-CHO+PRO, high-molecular-weight carbohydrate plus protein.

## Data Availability

The data presented in this study are available from the corresponding author upon reasonable request. The data are not publicly available due to participant privacy and ethical considerations.
